# DNA methylation patterns associated with oxidative stress in an ageing population

**DOI:** 10.1186/s12920-016-0235-0

**Published:** 2016-11-25

**Authors:** Åsa K. Hedman, Mihkel Zilmer, Johan Sundström, Lars Lind, Erik Ingelsson

**Affiliations:** 1 Department of Medical Sciences, Molecular Epidemiology and Science for Life Laboratory, Uppsala University, Uppsala, Sweden; 2 Institute of Biomedicine and Translational Medicine, Department of Biochemistry, The Centre of Excellence for Genomics and Translational Medicine, University of Tartu, Tartu, Estonia; 3 Department of Medical Sciences, Cardiovascular Epidemiology, Uppsala University Hospital, Uppsala, Sweden; 4 Department of Medicine, Division of Cardiovascular Medicine, Stanford University School of Medicine, Stanford, CA USA

**Keywords:** DNA methylation, Epigenetics, Oxidative stress, Type 2 diabetes, Cardiovascular disease

## Abstract

**Background:**

Oxidative stress has been related to type 2 diabetes (T2D) and cardiovascular disease (CVD), the leading global cause of death. Contributions of environmental factors such as oxidative stress on complex traits and disease may be partly mediated through changes in epigenetic marks (e.g. DNA methylation). Studies relating differential methylation with intermediate phenotypes and disease endpoints may be useful in identifying additional candidate genes and mechanisms involved in disease.

**Methods:**

To investigate the role of epigenetic variation in oxidative stress marker levels and subsequent development of CVD and T2D, we performed analyses of genome-wide DNA methylation in blood, ten markers of oxidative stress (total glutathione [TGSH], reduced glutathione [GSH], oxidised glutathione [GSSG], GSSG to GSH ratio, homocysteine [HCY], oxidised low-density lipoprotein (oxLDL), antibodies against oxLDL [OLAB], conjugated dienes [CD], baseline conjugated dienes [BCD]-LDL and total antioxidant capacity [TAOC]) and incident disease in up to 966 age-matched individuals.

**Results:**

In total, we found 66 cytosine-guanine (CpG) sites associated with one or more oxidative stress markers (false discovery rate [FDR] <0.05). These sites were enriched in regulatory regions of the genome. Genes annotated to CpG sites showed enrichment in annotation clusters relating to phospho-metabolism and proteins with pleckstrin domains. We investigated the contribution of oxidative stress-associated CpGs to development of cardiometabolic disease. Methylation variation at CpGs in the 3'-UTR of *HIST1H4D* (cg08170869; histone cluster 1, H4d) and in the body of *DVL1* (cg03465880; dishevelled-1) were associated with incident T2D events during 10 years of follow-up (all permutation p-values <0.01), indicating a role of epigenetic regulation in oxidative stress processes leading to development or progression of diabetes. Methylation QTL (meQTL) analysis showed significant associations with genetic sequence variants in *cis* at 28 (42%) of oxidative stress phenotype-associated sites (FDR < 0.05). Integrating *cis*-meQTLs with genotype-phenotype associations indicated that genetic effects on oxidative stress phenotype at one locus (cg07547695; *BCL2L11*) may be mediated through DNA methylation.

**Conclusions:**

In conclusion, we report novel associations of DNA methylation with oxidative stress, some of which also show evidence of a relation with T2D incidence.

**Electronic supplementary material:**

The online version of this article (doi:10.1186/s12920-016-0235-0) contains supplementary material, which is available to authorized users.

## Background

CVD is the most common cause of death globally. Risk factors of CVD include high blood pressure, smoking, hyperglycaemia, T2D and obesity [1]. Previous studies have suggested that increased oxidative stress as a consequence of obesity and T2D may contribute to the increased risk of CVD [2, 3]. Furthermore, CVD and risk factors of CVD have been associated with changes in levels of oxidative stress markers [4, 5].

Some of the oxidative markers have also been associated with sub-phenotypes of CVD and T2D. For example, oxLDL, elevated in CVD [6, 7], plays a role in atherosclerosis through its role in maturation of macrophages contributing to inflammation and foam formation [8, 9]. Furthermore, oxLDL is associated with insulin resistance [10] indicating a role of oxidative stress in progression to insulin resistance and T2D. The amino acid HCY has been associated with inflammation in blood vessels, progression to atherosclerosis and development of CVD, particularly ischemic stroke [11, 12]; however, the causal role of homocysteine has been challenged [13–15].

While genome-wide association studies (GWAS) have been successful in identifying numerous common genetic sequence variants associated with metabolic disease and CVD [16–20], so far these only explain a small proportion of the variability of these phenotypes. In addition, environmental factors also influence disease susceptibility. Such contributions may be partly mediated through changes in epigenetic marks (e.g. DNA methylation), affecting transcription through mechanisms independent of DNA sequence [21]. Thus, studies relating differential methylation with intermediate phenotypes and disease endpoints may be useful in identifying additional candidate genes and mechanisms involved in these diseases [22]. Previous studies support a role for DNA methylation in common complex diseases [23–26] and in mediation of environmental exposures of importance for CVD and T2D, such as cigarette smoking [27, 28] and oxidative stress [29, 30].

In this study, we aimed to examine epigenetic variation in blood cells in relation to oxidative stress and development of T2D and CVD. Blood-derived cells play a role in several processes relating to cardiometabolic disease [31, 32]. Furthermore, previous studies have shown methylation variation in blood to reflect differential methylation in various tissues [33–37]. We performed analyses of genome-wide DNA methylation, ten markers of oxidative stress (TGSH, GSH, GSSG, GSSG/GSH ratio, HCY, oxLDL, OLAB, CD, BCD-LDL and TAOC) and incident disease in 966 individuals from the general population.

## Methods

### Study sample

The Prospective Investigation of the Vasculature in Uppsala Seniors (PIVUS) is a prospective community-based cohort of participants from Uppsala, Sweden. All men and women at age 70 living in Uppsala in 2001 were invited to participate. The 1016 participants (50% women) have been extensively phenotyped, as described previously [38], and on the Internet (www.medsci.uu.se/pivus/). The participants have been re-examined at ages 75 and 80, and their morbidity and mortality has been followed via national registers and journal review. Clinical diagnoses by journal review of CVD and/or T2D at 80 years (10 years after baseline) were used to define disease events. For analysis of CVD outcomes, we included myocardial infarction (ICD-10 code: I21), stroke (ICD-10 code:I63) and heart failure (ICD-10 code: I50). During the 10 year follow up period (between ages 70 and 80), there were 142 deaths; 34 of these due to CVD related disease.

### Markers of oxidative stress

The methods of collection and validation of oxidative markers have previously been described [5]. Briefly, TGSH, GSH, CD and TAOC were determined using a method described and validated in Annuk et al. [39]. HCY levels were measured using an Enzyme Immunoassay method (Axis-Shield Diagnostics Ltd, UK). BCD-LDL were measured using a method described in detail in [40]. Enzyme-linked immuno-absorbent assays were used to determine levels of serum oxLDL (Mercodia AB, Sweden) and OLAB (BioMedica, Austria).

### Genome-wide DNA methylation profiling

Blood for the DNA methylation assay was collected at the baseline examination. Genomic DNA was extracted from blood samples and bisulphite conversion of 500 ng genomic DNA was performed using the EZ-96 DNA Methylation Gold Kit (Zymo Research Product, Germany). The equivalent of approximately 200 ng of bisulphite converted DNA was removed, evaporated to a volume of < 4 μl, and used for methylation profiling using the Illumina Infinium assay and the Illumina HumanMethylation450_v.1.2 bead chip according to the protocol from the supplier (Illumina Inc., San Diego, CA, USA). The results were analysed with GenomeStudio 2011.1 (Illumina Inc., San Diego, CA, USA). After exclusion of replicates, a total of 1002 study participants had methylation data available for quality control procedures. Three samples were excluded based on poor bisulphite conversion efficiency, twelve samples due to low pass rate of CpG sites (<98.5% with a detection *P*-value > 0.01) and a further six samples based on low SNP genotype match (>1 SNP mismatches) between genotypes from the methylation array and Omni/Metabochip genotyping chips leaving 981 samples. Following additional removal of participants with high leukocyte cell counts (>10x10^9^ cells/L; *n* = 14) and one individual with no data on oxidative stress markers, 966 individuals remained for downstream analysis. The signal intensities for the methylated and unmethylated states were then quantile normalised for each probe type separately, and beta values were calculated. Mapping and annotation of the 485,764 probes on the HumanMethylation450K BeadChip has previously been described [41]. Briefly, probes mapping to multiple locations (with at least two mismatches) in the human reference genome (GRCh37) were excluded leaving 459,433 uniquely mapping autosomal probes. Furthermore, probes were filtered based on sequence polymorphisms as follows: those with a common SNP (minor allele frequency [MAF] > 5%) within 10 bp of the methylation site and those overlapping copy number variants were excluded from analysis. This resulted in a final set of 455,127 probes which were then assigned to CpG islands and RefSeq transcripts downloaded from the UCSC Genome Browser (September, 2012). Probes within 2 kb away from borders of a CpG island were defined as shores and those within 2 kb of shores as falling within shelves. The rest were assigned to others/open sea. Probes were mapped in relation to transcripts as follows: TSS1500 (1500 bp to 200 bp upstream of transcriptional start site [TSS]), TSS200 (200 bp upstream of TSS), the 5'-UTR, the first exon, the gene body or the 3'-UTR [42].

### Genotyping and imputation

Individuals were genotyped using the Illumina OmniExpress and Illumina Metabochip microarrays. Prior to imputation, quality control was performed as described below. Exclusion of samples were performed based on the following criteria: genotype call rate <95%; heterozygosity >3 standard deviations (SD); gender discordance; duplicated samples; identity-by-descent match; and ethnic outliers. Monomorphic SNPs; or SNPs with Hardy-Weinberg equilibrium *p*-value < 1E10-6; genotype call rate < 0.99 (SNPs with MAF <5%) or <0.95 (SNPs with MAF ≥ 5%); MAF < 1% were excluded from analysis. Data were imputed to the 1000G (version: March 2012) multi population reference panel using Impute v.2.2.2 [43]. A plot of the PIVUS data with the data from the multi - population reference panel are included in Additional file 1: Figure S1.

### Statistical analyses

#### Association of methylation of blood cell-derived DNA with phenotypes and disease outcomes

Transformed or raw phenotypes were used (details in Table 1). All models were adjusted for age, sex, batching (clinical visit date), bisulphite conversion efficiency mean (calculated from control probes), bisulphite conversion plate and predicted white cell counts (estimated from the DNA methylation data using the Houseman algorithm [44], as implemented in R package *minfi* for Illumina HumanMethylation450 [45], with reference data on sorted blood cell populations from Reinius et al. [46]). To determine whether BMI confounds the relationship between the oxidative marker and DNA methylation, we performed secondary models additionally adjusted for BMI for those oxidative markers that showed association with BMI in sex adjusted models (nominal *p*-value < 0.01). For continuous phenotypes, the associations between normalised DNA methylation beta values and phenotypes were modelled by a linear model, using R [47] and the *lm* function, fitted by maximum-likelihood assuming a normally distributed error term. For binary phenotypes (case/control), we fitted a logistic regression in R using the *glm* function (binomial family [link function, logit]), to model the association between standardised DNA methylation and case/control status. Disease was used as the outcome variable, and technical covariates (as above), age, sex, predicted white cell count and standardised methylation as independent variables as follows: Disease status (1/0) ~ standardised methylation + age + sex + predicted white cell counts + technical covariates. In secondary models, we also included BMI and smoking as covariates in the model. In all cases, a likelihood ratio test was used to assess the significance of the phenotype effect. The *p*-value of the phenotype effect in each model was calculated from the Chi-square distribution with 1 degree of freedom using -2log(likelihood ratio) as the test statistic. FDR were estimated based on Q-values [48]. For CVD and T2D outcomes, significance was assessed using permutations of case/control status (10,000 permutations). A permutation *p*-value of < 0.01 was considered as significant.

**Table 1:** Methylation sites associated with oxidative markers in blood

Phenotype	Number	Mean (SD)	Range	No. of CpG sites associated at FDR <0.05	No. of CpG sites associated at *P*-value <1.1E-7
TGSH (μg/ml)	958	905.0 (199.3)	440–1836	18	3
GSH (μg/ml)	957	829.9 (191.1)	398–1730	25	2
GSSG (μg/ml)	959	75.8 (35.7)	23–257	1	1
GSSG/GSH ratio	957	0.1 (0.06)	0.03–0.53	21	3
HCY (μmol/l)	966	10.6 (3.98)	3.4–40.7	9	6
*ln* OxLDL (U/l)	966	132.8 (47.7)	42–285	1	1
CD (μmol/l)	966	40.7 (11.1)	13.8–90.4	1	1
BCD-LDL (μmol/l)	964	21.7 (7.4)	7.8–71.7	6	4
*ln* OLAB (U/l)	734	5.9 (0.96)	3.4–7.9	0	0
TAOC (%)	957	37.6 (3.9)	22–55	0	0

*SD* standard deviation, *CpG* cytosine-guanine site, *FDR* false discovery rate, *TGSH* total glutathione level, *GSH* reduced glutathione, *GSSG* oxidised glutathione, *HCY* homocysteine, *OxLDL* oxidised LDL, *CD* conjugated dienes, *BCD* baseline CD, *OLAB* antibodies against oxLDL, *TAOC* total antioxidant capacity

#### meQTL and SNP-phenotype analyses

Associations between normalised DNA methylation beta values and genotypes were modelled by a linear model, using R [47] and the *lm* function, fitted by maximum-likelihood assuming a normally distributed error term. We assumed an additive genetic model. A likelihood ratio test was used to assess the significance of the SNP effect. The *p*-value of the SNP effect in each model was calculated from the Chi-square distribution with 1 degree of freedom using -2log(likelihood ratio) as the test statistic. We only performed *cis* analysis, which was limited to SNPs located within 100 kb either side of the probe location. To perform analyses in R, genotype probabilities (from IMPUTE) were transformed to posterior mean genotypes (MACH format [49]). Further, we only included SNPs with a MAF <5% and INFO (from imputation) >0.8 in down-stream analyses. Since we did not perform a array-wide *cis*-meQTL scan, and were therefore concerned of potential bias in the *p*-value distribution, we estimated FDR from permutations, rather than using Q-values. We permuted SNP data, performed *cis*-meQTL analysis on the permuted data, and repeated this for ten replicates selecting the most associated SNP per methylation probe in each round of permutation. FDR of 0.05 was calculated as the nominal *p*-value threshold which gave less than 5% significant associations (i.e. false discoveries) in the permuted data. Associations between oxidative markers and meQTL genotypes were modelled by a linear model, using R [47] and the *lm* function, fitted by maximum-likelihood assuming a normally distributed error term. We assumed an additive genetic model. A likelihood ratio test was used to assess the significance of the SNP effect. The *p*-value of the SNP effect in each model was calculated from the Chi-square distribution with 1 degree of freedom using -2log(likelihood ratio) as the test statistic. Significance was assessed using permutations of genotype data (10,000 permutations). Using a one-sided Fisher exact test we tested for over representation of significant meQTL SNPs in nominally significant GWAS associations (*p*-value < 0.05) using GWAS data from the CARDIoGRAM consortium for CHD [20] and the DIAGRAM consortium for T2D [18].

#### Enrichment in genomic location, regulatory regions, transcription factor binding and biological processes

Using annotation data described above we tested whether CpGs associated with oxidative markers were enriched in genomic locations with respect to genes and CpG islands. Enrichment was assessed using a two-sided Fisher exact test. Overlap of associated CpGs with functional regulatory elements across cell types were assessed using data available at RegulomeDB [50]. We determined if the overlap was more than expected by chance by comparing this to random set of CpGs. To assess whether genes annotated to phenotype-associated CpGs are likely to be regulated by a common set of transcription factors (TFs), we utilised PSCAN [51] with the JASPAR database [52]. To place our data in the context of biological processes or pathways, we subjected genes annotated to CpGs associated with phenotypes or genotypes to pathway analysis using DAVID [53, 54] and PANTHER [55–57]. We used only genes on the array as background and considered terms with a *p*-value < 0.05 following adjustments for multiple testing (to the number of pathways or ontology terms) as significant.

## Results

### Identification of DNA methylation patterns of oxidative stress

**Fig. 1:**
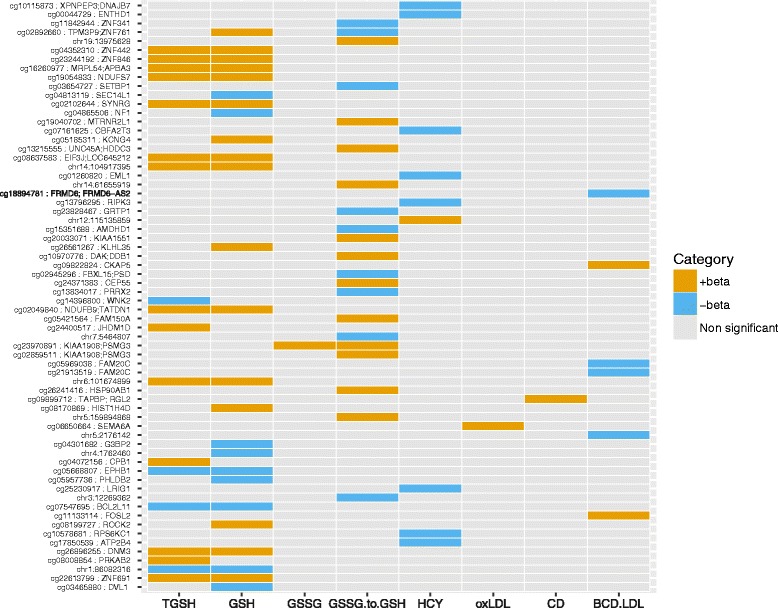
Overview of CpGs associated with oxidative markers (FDR < 0.05). CpGs are ordered by chromosomal position from bottom (chr. 1) to top (chr. 22). Two of the oxidative markers (OLAB and TAOC) showed no significant associations with CpGs and were not included in the figure

We characterised genome-wide blood DNA methylation patterns at 459,235 CpGs mapping uniquely across the genome in 966 70-year old individuals from the PIVUS cohort [38]. We performed genome-wide association scans to determine DNA methylation patterns associated with ten markers of oxidative stress: TGSH, GSH, GSSG, GSSG/GSH ratio, HCY, oxLDL, OLAB, CD, BCD-LDL and TAOC (Table 1). In total, we observed 66 CpGs for which levels of methylation were associated with one or more oxidative markers at a-per-trait FDR of <0.05; 18 of these were also associated with at least one oxidative marker at a Bonferroni-corrected alpha threshold <0.05 (taking the number of CpGs into account; ﻿Additional file 2: Figure S2). Figure 1 shows the associations of CpGs with oxidative stress markers.

#### Glutathione

Glutathione is an important antioxidant and the balance between the reduced and oxidised form is indicative of the oxidative state of an individual. We investigated the association between genome-wide methylation patterns and TGSH, GSH and GSSG and found 18, 25 and one significant CpGs, respectively (FDR < 0.05; Additional file 3: Table S1–S3). As should be expected, a large proportion of CpGs associated with TGSH and GSH was shared (14 CpGs; Fig. 1), including sites annotated to *BCL2L11* (cg07547695; BCL2-like 11 [apoptosis facilitator]), *NDUFS7* (cg19054833; NADH dehydrogenase [ubiquinone] Fe-S protein 7, 20 kDa) and *EIF3J* (cg08637583; eukaryotic translation initiation factor 3, subunit J). The ratio of GSSG to GSH can be used as a marker of oxidative stress. We found methylation at 21 CpGs to be significantly associated with GSSG/GSH ratio (FDR < 0.05; Additional file 3: Table S4). Methylation in the promoter region of *ZNF761* (cg02892660; zinc finger protein 761) and *PSMG3* (cg23970891; proteasome assembly chaperone 3), showed the strongest associations with GSSG/GSH ratio.

#### Homocysteine

In our analysis, methylation at nine CpGs were significantly associated with levels of the amino acid HCY (FDR < 0.05; Additional file 3: Table S5), including sites annotated to genes with a role in signal transduction (*ENTHD1* [cg00044729], *LRIG1*[cg25230917], *RIPK3* [cg13796295], *RPS6KC1* [cg10578681], *ATP2B4* [cg17850539]) and lipid-related processes (*RPS6KC1* [cg10578681]).

#### Lipid-related oxidative markers

In genome-wide methylation analysis, oxLDL was associated with methylation in the 5'-UTR of *SEMA6A* (cg06650664; sema domain, transmembrane domain [TM], and cytoplasmic domain, [semaphorin] 6A), a protein with anti-angiogenic function [58, 59] (FDR < 0.05; Additional file 3: Table S6). Oxidation of LDL can also be monitored by markers CD and BCD-LDL which measure products of lipid peroxidation. In our analysis, CD was found to associate with higher methylation at a CpG site located in the 3'-UTR of *TAPBP* (cg09899712; TAP binding protein [tapasin]) and the promoter region (TSS1500) of *RGL2* (cg09899712; ral guanine nucleotide dissociation stimulator-like 2 (FDR < 0.05; Additional file 3: Table S7). Methylation levels at six CpGs were associated with BCD-LDL (FDR < 0.05; Additional file 3: Table S8). These were annotated to *CKAP5* (cg09822824; cytoskeleton associated protein 5), *FOSL2* (cg11133114; FOS-like antigen 2), *FAM20C* (cg21913519, cg05969038; family with sequence similarity 20, member C) and *FRMD6* (cg18894781; FERM Domain containing 6). We observed no overlap in significant CpGs between the three lipid-related oxidative markers. As CD (*p*-value = 0.00011) and BCD-LDL (*p*-value = 0.0091) were found to be associated with BMI in sex-adjusted models in our study, secondary models adjusted for BMI were also performed for these two phenotypes (Additional file 3: Tables S7–S8). None of the phenotype-associated CpGs showed a large change in the regression coefficient for the oxidative marker after adjustment with BMI, indicating that BMI did not confound the relationship between DNA methylation and these oxidative markers (Additional file 1: Figure S3).

### Functional characterisation of methylation sites associated with oxidative stress

**Fig. 2:**
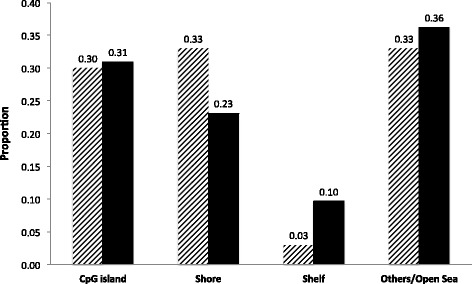
CpG island context of CpGs associated with oxidative markers. CpGs were classified into: CpG island, Shore, Shelf and Others/Open sea, and phenotype-associated CpGs (*striped bars*) were compared with all CpGs on the array (*black bars*). *P*-values in figure represent results of a one-sided Fisher exact test testing for over- or under-representation of term in either group

**Fig. 3:**
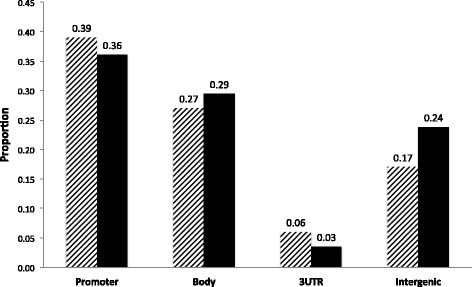
Genomic distribution of CpGs associated with oxidative markers. CpGs were classified into: promoter (TSS1500, TSS200, 5'-UTR, First exon), Body, 3'-UTR and intergenic. Phenotype-associated CpGs (*striped bars*) were compared to all CpGs on the array (*black bars*). *P*-values in figure represent results of a one-sided Fisher exact test testing for over- or under-representation of term in either group

We explored the functional role of CpGs associated with oxidative markers through investigation of their genomic location with respect to genes, CpG islands and functional regulatory elements. Phenotype-associated CpGs were enriched in CpG island shores compared to all CpGs on the array (enrichment *p*-value = 0.04, Fig. 2). A larger proportion of phenotype-associated CpGs (as compared to all CpGs) were located in promoters of genes (45% vs. 36%; enrichment *p*-value = 0.06, Fig. 3); thus having the potential to affect transcription from adjacent genes. We assessed the overlap of associated CpGs with functional regulatory elements across cell types using RegulomeDB [50]. Twenty-three percentages of sites showed strong evidence of being located in a functional regulatory region (RegulomeDB score 1a-2c; Additional file 3: Tables S1–S8). This was more than expected by chance (permutation *p*-value = 0.01).

To assess whether genes annotated to phenotype-associated CpGs are likely to be regulated by a common set of TFs, we used PSCAN [51] with the JASPAR database [52]. We found significant enrichment of four TF binding sites (adjusted *p*-value <0.05; Additional file 1: Table S9), including binding sites of E2F1, a gene/protein with a potential role in atherosclerosis and coronary heart disease (CHD) [60].

To further place our findings in biological context, we performed gene set enrichment analysis [55–57] on the 64 genes annotated to CpGs associated with oxidative markers. Using the *Functional Annotation Clustering Tool* in DAVID [53], we found evidence of genes clustering into annotations (enrichment score > 1) relating to functional terms phosphorylation and phosphate/phosphorous metabolic processes and pleckstrin domains (Additional file 1: Table S11).

### Genetic associations of oxidative stress-associated DNA methylation

Genetic sequence variants have been shown to contribute to DNA methylation variation, so called meQTLs [61]. We examined the presence of such loci for the 66 CpGs associated with oxidative markers, and found 28 (41.8%) of phenotype-CpGs with a significant *cis*-meQTL (FDR < 0.05, nominal *p*-value <2.07E-4, Additional file 1: Table S12). To assess whether genetic associations with markers of oxidative stress may be mediated by epigenetics, we examined whether genotype-phenotype, genotype-CpG and CpG-phenotype associations overlapped (an overview of the analysis are available in Additional file 4: Figure S4). Overall, we investigated whether *cis*-meQTL SNPs associated with the oxidative markers and found modest suggestive evidence of association (*p*-value <0.001) for 171 SNPs (0.56%) with at least one of the oxidative markers. We found two instances (at cg07547695 in the 5'-UTR of *BCL2L11*, associated with both TGSH and GSH) for which associations of genotype-phenotype (*p*-value <0.001), *cis*-meQTL (FDR < 0.05) and CpG-phenotype (FDR < 0.05) overlapped (Additional file 1: Table S13; Additional file 4: Figure S4). A proxy of rs6750142 (rs726430, *r* ^*2*^ = 1) associated with methylation at cg07547695 was nominally associated (*p*-value <0.05) with both CHD and T2D in GWAS [18, 20].

We investigated the behaviour of 1599 significant meQTL SNPs (FDR < 0.05) in GWAS data from the CARDIoGRAM consortium for CHD [20] and the DIAGRAM consortium for T2D [18]. We found no evidence of overrepresentation of nominally significant associations (*p*-value <0.05) for CHD (one-sided Fisher exact *p*-value = 0.99) or T2D (one-sided Fisher exact *p*-value = 0.93) among significant meQTL SNPs. One or more significant meQTL SNPs of nine CpG sites were nominally associated with CHD in GWAS data from the CARDIoGRAM consortium (*p*-value < 0.05). These were annotated to *AMDHD1* (cg15351688: rs10777751, rs7955450, rs7486703), *BCL2L11* (cg07547695: rs726430), *FAM20C* (cg05969038: rs7786461; cg21913519: rs7786461), *HSP90AB1* (cg26241416: rs6905285, rs7758726, rs666462), *SEMA6A* (cg06650664: rs10077506, rs17139825), *WNK2* (cg14396800: rs2991377,rs10992689, rs10821105) and intergenic sites on chr. 5 (cg15609272: rs17057846) and chr. 12 (cg17173663: rs1354156) (Additional file 5). In analogous analysis on GWAS data from the DIAGRAM consortium, we found nominal associations for SNPs associated with methylation at CpG sites annotated to *BCL2L11* (cg07547695: rs726430), *CEP55* (cg24371383: rs12782691), *CPB1* (cg04072156: rs16861015) and an intergenic site on chr. 4 (cg14532755: rs1665364, rs2236786, rs3752749, rs732754, rs744658, rs798719, rs798726, rs798727, rs798741, rs798744, rs798751, rs798754, rs798755, rs798756, rs798766, rs811316, rs8389) (*p*-value < 0.05; Additional file 5).

### The relationship between oxidative stress-associated CpGs and disease incidence

As markers of oxidative stress have been associated with both CVD and T2D, we sought to assess the role of epigenetics in this process. Methylation levels at the 66 CpGs associated with oxidative markers were tested for association with incident CVD (*n* = 180) and T2D (*n* = 71) events during a 10-year follow-up using logistic regression models to examine their potential role in disease. Epigenetic variation in the 3'-UTR of *HIST1H4D* (cg08170869; histone cluster 1, H4d) and in the body of *DVL1* (cg03465880; dishevelled-1) were associated with incident T2D (permutation *p*-value <0.01; Table 2). For every SD decrease in methylation β value at cg08170869, the risk of T2D was 39% higher (nominal *p*-value = 0.0034; permutation *p*-value = 0.0044; odds ratio [OR] per SD decrement = 1.39 [95% CI, 1.15–1.57]). Similarly, hypomethylation in *DVL1* was associated with higher risk of T2D (nominal *p*-value = 0.0073; permutation *p*-value = 0.0080; OR per SD decrement = 1.31 [95% CI, 1.10–1.47]).

**Table 2:** Association of oxidative marker-associated CpGs with incident T2D^a^ events (permutation *p*-value < 0.01)

					Primary model			Secondary model^d^	
Disease outcome	CpG	Gene	Description	Gene Property	OR (95% CIs)^b^	Nominal *P*-value	Permutation *P*-value^c^	*P*-value	Oxidative marker association (Direction)^e^
T2D	cg08170869	*HIST1H4D*	Histone cluster 1, H4d	3'-UTR	1.39 (1.15–1.57)	3.39E-03	4.40E-03	9.19E-03	GSH (+)
T2D	cg03465880	*DVL1*	Dishevelled segment polarity protein 1	Body	1.31 (1.10–1.47)	7.27E-03	8.00E-03	6.48E-03	GSH (−)

*T2D* type 2 diabetes, *CpG* cytosine-guanine site, OR odds ratio, CI confidence intervals, GSH reduced glutathione, *SD* standard deviation

^a^Events up to 10 years after baseline; number of T2D events = 71

^b^Corresponds to the OR increase in risk of disease per SD decrement in DNA methylation

^c^ *P*-value from permutation test (*n* = 10,000 permutations)

^d^Secondary model also included BMI and smoking

^e^Direction of association between oxidative marker(s) and methylation at CpG site. A + symbol corresponds to that increased levels of DNA methylation associate with increased levels of the oxidative marker

Analogous analysis of CVD revealed no significant association of methylation levels at the phenotype-associated CpGs with incident CVD events (permutation *p*-value <0.01). The strongest association was to methylation levels at cg11842944 in the body of *ZNF341* (nominal *p*-value = 0.013; permutation *p*-value = 0.016; OR per SD decrement = 1.23 [95% CI, 1.05–1.42]). Analysing myocardial infarction (*n* = 55), heart failure (*n* = 76) and ischaemic stroke (*n* = 47) separately, methylation at this CpG showed the strongest association to heart failure (nominal *p*-value = 0.0087; permutation *p*-value = 0.0126; OR per SD decrement = 1.28 [95% CI, 1.06–1.50]). This gene lies in a region previously associated with height in GWAS [62] and encodes a gene product involved in transcriptional regulation.

## Discussion

Oxidative stress has previously been associated with development of cardiometabolic disease. In this study, the role of epigenetic changes in blood cells for oxidative stress and development of CVD and T2D was examined through analyses of genome-wide DNA methylation data and ten markers of oxidative stress, CVD and T2D in up to 966 individuals of the same age.

We identified numerous blood CpGs for which levels of methylation correlated with markers of oxidative stress. Enrichment of associations to DNA methylation in CpG island shores, previously noted to be dynamic [63, 64] and correlated with gene expression [63], indicates that differential methylation with oxidative stress may play a role in transcriptional regulation. Overlap with functional regulatory elements for one-fourth of associated CpGs support a functional role for these methylation changes with oxidative stress. Enrichment of TF binding sites in regions upstream of genes annotated to phenotype-associated CpGs indicates a common set of regulatory signals. TFs included E2F1 with prior evidence of a function in processes relating to atherosclerosis and CHD [60], connecting genes for which CpG methylation changed with oxidative stress to CVD.

Methylation variation in the 3'-UTR of *HIST1H4D* (cg08170869) and in the body of *DVL1* (cg03465880) associated with GSH were also associated with incident T2D. Previous evidence indicates changes in levels of GSH in individuals with T2D [65, 66]. Evidence from previous studies implicates *DVL1* in processes related to T2D [67]. *DVL1* encodes a gene product with an important role in Wnt signalling, important in adipogeneis, and has, for example, been found to be down-regulated in adipocytes from non-obese insulin resistant individuals compared to controls [67].

Roughly forty percent of oxidative stress-associated CpGs were regulated by genetic sequence variation in *cis*. We found evidence of genotype-phenotype associations acting via epigenetic variation at gene *BCL2L11* (BCL2-like 11 [apoptosis facilitator]). Genetic variants close to this gene have previously been associated with the biological ageing marker dehydroepiandrosterone sulphate in GWAS [68]. Integration of meQTLs with GWAS data on CHD and T2D showed no significant enrichment in nominal associations for meQTL SNPs of oxidative stress-associated CpGs.

Previous evidence indicates a role for some of the genes annotated to oxidative stress marker-associated CpGs in metabolic or cardiovascular disease, indicating that epigenetic changes with oxidative stress may reflect important disease processes. Lipid-related oxidative markers such as oxLDL has previously been shown to play a role in atherosclerosis [8, 9] and to associate with insulin resistance [10]. In agreement with this, we found methylation at genes involved in some of these processes to associate with lipid-related oxidative markers. *RGL2* (for which promoter methylation was higher with higher CD) has been shown to have a protective role in response to cardiac stress in vitro [69]. Furthermore, previous results implicate RGL2 in atherosclerosis pathogenesis. RGL2 in complex with SAMD9 have an inhibitory function on expression of the transcription factor *EGR1*, which is highly expressed in atherosclerotic lesions and has been shown to be involved in induction of the coagulation protein tissue factor in response to oxLDL [70–72]. Earlier studies suggest a role of the transcription factor FOSL2 (for which intragenic methylation was higher with BCD-LDL) in processes relating to cardiac fibrosis of ischaemic tissue through its oxygen-sensitive induction of TGFβ in cardiac fibroblasts [73]. FOSL2 also regulates leptin expression in adipocytes [74]. The association of BCD-LDL to promoter methylation of this gene in blood was not driven by obesity as the signal still remained following adjustment for BMI. Additionally, genetic studies of QRS duration [75], which has been associated with increased risk of heart failure [76, 77], indicate a role of *LRIG1*. In our study, methylation at this gene associated with HCY, an amino acid previously associated with inflammation in blood vessels, progression to atherosclerosis and development of CVD, particularly ischemic stroke [11, 12]. However, the causal role of homocysteine in CVD has been challenged [13–15].

The main strengths of the present study include the large sample size that underwent measurements of genome-wide DNA methylation, ten markers of reflecting different aspects of oxidative stress and the 10 years of follow-up allowing analyses of incident disease endpoints. The study also has some limitations. First, we acknowledge that the most important limitation is the lack of replication of oxidative stress-associated DNA methylation. To our knowledge, there are no other study samples with the needed data available making replication impossible, but we have done our best to avoid false positive findings via strict correction for multiple testing and integration with data on gene function, regulation and on related phenotypes from external data sources. Second, gene expression data for the same individuals were not available to assess the effect of epigenetic variation with oxidative markers on transcription in blood. Third, while blood is easily accessible and thus attractive for biomarker discovery, clinical diagnostics and translation, blood derived cells may not be the most relevant tissue for drawing biological conclusions about oxidative stress, CVD and T2D. However, previous studies have shown methylation variation in blood to be a good proxy of differential methylation in various tissues [33–37].

## Conclusions

We found novel epigenetic changes in blood to be associated with markers of oxidative stress, two of these with evidence of a relation to T2D. Further studies are needed to replicate the findings on DNA methylation with oxidative stress, as well as determining the effect of epigenetic variation related to oxidative stress on downstream molecular biological phenotypes.

## Additional files

Additional file 1: Supplementary Data. **Figure S1.** Plot showing the first two PC components of the PIVUS genotype data with the 1000G multi population reference panel. **Figure S3.** Comparison of regression coefficients from the primary and secondary models (additionally adjusted for BMI) for oxidative marker BCD-LDL. **Table S9.** Enrichment in JASPAR transcription factor binding site motifs in genes annotated to oxidative stress associated CpGs (Bonferroni-adjusted *p*-value < 0.05). **Table S10.** Enriched biological process among genes annotated to oxidative marker associated CpGs (adjusted *p*-value < 0.05). **Table S11.** Enriched annotation clusters among genes annotated to oxidative marker CpGs (enrichment score > 1). **Table S12.** Significant lead *cis*-meQTL SNPs of oxidative marker CpGs (FDR <0.05). **Table S13.** Overlap across genotype-CpG (FDR <0.05), genotype-phenotype (*p*-value <0.001), and CpG-phenotype (FDR <0.05) results. (DOCX 135 kb)
Additional file 2: Figure S2. DNA methylation sites associated with oxidative markers at a Bonferroni-corrected alpha threshold 0.05 (*p*-value <1.1E-07). CpG sites are ordered by chromosomal position from bottom (chr. 1) to top (chr. 22). (EPS 14 kb)
Additional file 3: Supplemental Tables. **Table S1.** Methylation sites associated with TGSH (FDR <0.05). **Table S2.** Methylation sites associated with GSH (FDR <0.05. **Table S3.** Methylation sites associated with GSSG (FDR <0.05). **Table S4.** Methylation sites associated with ratio of GSSG-to-GSH (FDR <0.05). **Table S5.** Methylation sites associated with levels of HCY (FDR <0.05). **Table S6.** Methylation sites associated with levels of oxLDL (FDR <0.05). **Table S7.** Methylation sites associated with levels of CD (FDR <0.05). **Table S8**: Methylation sites associated with BCD-LDL (FDR <0.05). (XLS 185 kb)
Additional file 4: Figure S4. Overview of the analysis of genetic associations of oxidative stress-associated DNA methylation. The diagram shaded in blue depicts the associations at the two instances where we observed an overlap across genotype-CpG (FDR < 0.05), genotype-phenotype (p < 0.001), and CpG-phenotype (FDR < 0.05). (XLSX 60 kb)
Additional file 5: Table S14. Nominal significant associations (*p*-value <0.05) for significant meQTL SNPs in GWAS data from the CARDIOGRAMplusC4D and DIAGRAM consortia. (XLSX 59 kb)

## Abbreviations

BCD-LDL: Baseline conjugated dienes LDL
BMI: Body mass index.
CD: Conjugated dienes
CHD: Coronary heart disease
CpG: Cytosine-guanine
CVD: Cardiovascular disease
FDR: False discovery rate
GSH: Reduced glutathione
GSSG: Oxidised glutathione
GWAS: Genome-wide association studies
HCY: Homocysteine
LDL: Low-density lipoprotein
MAF: Minor allele frequency
meQTL: Methylation quantitative trait loci
OLAB: Antibodies against oxLDL
oxLDL: Oxidised LDL
PIVUS: The Prospective Investigation of the Vasculature in Uppsala Seniors
SD: Standard deviation
T2D: Type 2 diabetes
TAOC: Total antioxidant capacity
TGSH: Total glutathione
TSS: Transcriptional start site
