# Supplementary material for: DNA methylation patterns associated with oxidative stress in an ageing population
Source: BMC Med Genomics. 2016 Nov 25;9:72. doi: 10.1186/s12920-016-0235-0 (PMC5123374; doi:10.1186/s12920-016-0235-0)
Supplement: Additional file 1: — Supplementary Data. Figure S1. Plot showing the first two PC components of the PIVUS genotype data with the 1000G multi population reference panel. Figure S3. Comparison of regression coefficients from the primary and secondary models (additionally adjusted for BMI) for oxidative marker BCD-LDL. Table S9. Enrichment in JASPAR transcription factor binding site motifs in genes annotated to oxidative stress associated CpGs (Bonferroni-adjusted p-value < 0.05). Table S10. Enriched biological process among genes annotated to oxidative marker associated CpGs (adjusted p-value < 0.05). Table S11. Enriched annotation clusters among genes annotated to oxidative marker CpGs (enrichment score > 1). Table S12. Significant lead cis-meQTL SNPs of oxidative marker CpGs (FDR <0.05). Table S13. Overlap across genotype-CpG (FDR <0.05), genotype-phenotype (p-value <0.001), and CpG-phenotype (FDR <0.05) results. (DOCX 135 kb) [file 12920_2016_235_MOESM1_ESM.docx]

## Additional file 3: Supplementary data

Contents

| **Figure S1:** Plot showing the first two PC components of the PIVUS genotype data with the 1000G multi population reference panel | Page 2 |
| --- | --- |
| **Figure S3:** Comparison of regression coefficients from the primary and secondary models (additionally adjusted for BMI) for oxidative marker BCD-LDL | Page 3 |
| **Table S9:** Enrichment in JASPAR transcription factor binding site motifs in genes annotated to oxidative stress associated CpGs (Bonferroni-adjusted p-value < 0.05). | Page 4 |
| **Table S10:** Enriched biological process among genes annotated to oxidative marker associated CpGs (adjusted p-value < 0.05) | Page 5 |
| **Table S11:** Enriched annotation clusters among genes annotated to oxidative marker CpGs (enrichment score > 1) | Page 5 |
| **Table S12:** Significant lead *cis*-meQTL SNPs of oxidative marker-CpG sites (FDR<0.05). | Page 6 |
| **Table S13:** Overlap across genotype-CpG (FDR<0.05), genotype-phenotype (p<0.001), and CpG-phenotype (FDR<0.05) results | Page 7 |

**Figure S1: Plot showing the first two PC components of the PIVUS genotype data with the 1000G multi population reference panel.** Included are data from PIVUS (in blue) and from the 1000G multi population reference panel, showing that, as expected, PIVUS are most similar to the European Ancestry population (CEU).

**Figure S3: Comparison of regression coefficients from the primary and secondary models (additionally adjusted for BMI) for oxidative marker BCD-LDL**

**Table S9:** Enrichment in JASPAR transcription factor binding site motifs in genes annotated to oxidative stress associated CpGs (Bonferroni-adjusted p-value < 0.05).

| **TF Name** | **Matrix ID** | **Description** | **Nominal p-value** | **Adjusted p-value** |
| --- | --- | --- | --- | --- |
| TFAP2A(var.2) | MA0810.1 | Transcription factor AP-2 alpha (activating enhancer binding protein 2 alpha) | 4.12E-06 | 2.61E-03 |
| TFAP2B | MA0811.1 | Transcription factor AP-2 beta (activating enhancer binding protein 2 beta) | 6.01E-06 | 3.81E-03 |
| TFAP2C | MA0524.2 | Transcription factor AP-2 gamma (activating enhancer binding protein 2 gamma) | 9.23E-06 | 5.86E-03 |
| E2F1 | MA0024.2 | E2F transcription factor 1 | 1.23E-05 | 7.82E-03 |

**Table S11:** Enriched annotation clusters among genes annotated to oxidative marker CpGs (enrichment score > 1)

| **Term** | **Genes** |
| --- | --- |
| **Annotation Cluster 1: Enrichment Score 1.31** | |
| GO:0016310~phosphorylation | *NDUFS7, ROCK2, NDUFB9, RIPK3, RPS6KC1, WNK2, EPHB1* |
| GO:0006793~phosphorus metabolic process | *NDUFS7, ROCK2, NDUFB9, RIPK3, RPS6KC1, WNK2, EPHB1* |
| GO:0006796~phosphate metabolic process | *NDUFS7, ROCK2, NDUFB9, RIPK3, RPS6KC1, WNK2, EPHB1* |
| **Annotation Cluster 2: Enrichment Score 1.20** | |
| IPR001849:Pleckstrin homology | *DNM3, PSD, ROCK2, PHLDB2* |
| SM00233:PH | *DNM3, PSD, ROCK2, PHLDB2* |
| domain:PH | *DNM3, PSD, ROCK2, PHLDB2* |

**Table S12:** Significant lead *cis*-meQTL SNPs of oxidative marker-CpG sites (FDR<0.05).

| **ProbeID** | **Chr.** | **Position (b37)** | **Strand** | **Gene Property** | **Gene** | **SNP** | **Position (b37)** | **A1/A2** | **BETA** | **SE** | **P-value** | **Trait** | **Direction** |
| --- | --- | --- | --- | --- | --- | --- | --- | --- | --- | --- | --- | --- | --- |
| cg17850539 | 1 | 203708912 | + | Body/3UTR | *ATP2B4* | chr1:203623104:D [rs147934279] | 203623104 | TATATATATA/T | 0.0084 | 0.0022 | 1.09E-04 | HCY | **-** |
| cg10578681 | 1 | 213223453 | + | TSS1500 | *RPS6KC1* | rs56132207 | 213131477 | C/T | -0.0168 | 0.0031 | 4.50E-08 | HCY | **-** |
| cg07547695 | 2 | 111881075 | + | 5UTR | *BCL2L11* | rs72837815 | 111915068 | C/T | -0.0084 | 0.0018 | 1.70E-06 | TGSH, GSH | **-,-** |
| cg04399631 | 3 | 12269362 | - | -- | *intergenic* | rs13066322 | 12269266 | C/T | 0.0125 | 0.0019 | 3.69E-11 | GSSG/GSH | **-** |
| cg04072156 | 3 | 148544962 | - | TSS1500 | *CPB1* | rs7615559 | 148544932 | C/T | -0.0126 | 0.0013 | 6.90E-22 | TGSH | **+** |
| cg14532755 | 4 | 1762460 | + | -- | *intergenic* | rs13110202 | 1761205 | G/A | 0.0250 | 0.0035 | 1.36E-12 | GSH | **-** |
| cg08496086 | 5 | 2176142 | + | -- | *intergenic* | rs72710657 | 2144278 | T/G | -0.0069 | 0.0018 | 1.82E-04 | BCD-LDL | **-** |
| cg06650664 | 5 | 115870174 | + | 5UTR | *SEMA6A* | rs2303752 | 115785512 | C/T | -0.0066 | 0.0014 | 1.41E-06 | oxLDL | **+** |
| cg15609272 | 5 | 159894868 |  | -- | *intergenic* | rs77701841 | 159989800 | C/T | 0.0095 | 0.0018 | 1.19E-07 | GSSG/GSH | **+** |
| cg26241416 | 6 | 44215763 | - | 5UTR | *HSP90AB1* | rs324128 | 44212311 | T/C | 0.0213 | 0.0008 | 3.46E-172 | GSSG/GSH | **+** |
| cg21913519 | 7 | 209058 | - | Body | *FAM20C* | rs3814456 | 149628 | A/G | -0.0142 | 0.0022 | 2.02E-10 | BCD-LDL | **-** |
| cg05969038 | 7 | 213500 | + | Body | *FAM20C* | rs3814456 | 149628 | A/G | -0.0181 | 0.0024 | 1.41E-13 | BCD-LDL | **-** |
| cg05421564 | 8 | 53477218 |  | Body | *FAM150A* | rs1371945 | 53487745 | A/T | 0.0161 | 0.0017 | 5.25E-21 | GSSG/GSH | **+** |
| cg14396800 | 9 | 95996796 | - | Body | *WNK2* | rs7032194 | 95993429 | G/C | -0.0080 | 0.0019 | 3.87E-05 | TGSH | **-** |
| cg14396800 | 9 | 95996796 | - | Body | *WNK2* | rs10992685 | 95993757 | G/A | -0.0080 | 0.0019 | 3.87E-05 | TGSH | **-** |
| cg13834017 | 9 | 132484151 | + | Body | *PRRX2* | rs10988477 | 132454520 | C/T | -0.0181 | 0.003 | 2.57E-09 | GSSG/GSH | **-** |
| cg24371383 | 10 | 95256104 |  | TSS1500 | *CEP55* | rs1880264 | 95256144 | C/A | 0.0492 | 0.0020 | 1.79E-140 | GSSG/GSH | **+** |
| cg15351688 | 12 | 96350648 | - | Body | *AMDHD1* | rs7486703 | 96337997 | A/G | 0.0110 | 0.0010 | 1.73E-27 | GSSG/GSH | **-** |
| cg17173663 | 12 | 115135859 | - | --- | *intergenic* | rs1463888 | 115136391 | T/G | -0.0091 | 0.0021 | 1.84E-05 | HCY | **+** |
| cg23828467 | 13 | 113992858 | - | Body | *GRTP1* | rs9577511 | 113991823 | A/G | 0.0105 | 0.0023 | 3.38E-06 | GSSG/GSH | **-** |
| cg13796295 | 14 | 24807658 | - | Body | *RIPK3* | rs2332232 | 24756284 | T/C | 0.0104 | 0.0027 | 1.11E-04 | HCY | **-** |
| cg05185311 | 16 | 84256194 | - | Body | *KCNG4* | rs244808 | 84266483 | G/T | 0.0026 | 0.0006 | 2.08E-05 | GSH | **+** |
| cg19040702 | 17 | 22023833 | - | 3UTR | *MTRNR2L1* | rs62051411 | 22023591 | T/C | 0.0083 | 0.0017 | 1.18E-06 | GSSG/GSH | **+** |
| cg19054833 | 19 | 1383453 | + | TSS1500 | *NDUFS7* | chr19:1298966:I | 1298966 | G/GT | 0.0023 | 0.0006 | 1.08E-04 | TGSH, GSH | **+, +** |
| cg23244192 | 19 | 9879544 | - | TSS200 | *ZNF846* | rs12462027 | 9926860 | G/A | -0.0042 | 0.0006 | 1.68E-13 | TGSH, GSH | **+, +** |
| cg04352310 | 19 | 12472690 | + | Body | *ZNF442* | rs12977116 | 12473577 | T/G | -0.0034 | 0.0008 | 6.34E-05 | TGSH, GSH | **+, +** |
| cg25388447 | 19 | 13975628 | - | -- | *intergenic* | rs61686002 | 14047684 | G/C | 0.0144 | 0.0035 | 4.17E-05 | GSSG/GSH | **+** |
| cg00044729 | 22 | 40290976 | - | TSS1500 | *ENTHD1* | rs137992 | 40344043 | A/G | -0.004 | 0.0011 | 1.84E-04 | HCY | **-** |
| cg10115873 | 22 | 41258407 | + | Body/3UTR; TSS1500 | *XPNPEP3; DNAJB7* | rs5758067 | 41161986 | C/G | -0.0067 | 0.0011 | 4.40E-09 | HCY | **-** |

**Table S13:** Overlap across genotype-CpG (FDR<0.05), genotype-phenotype (p<0.001), and CpG-phenotype (FDR<0.05) results.

|  |  |  |  |  |  |  |  |  | **Genotype - Phenotype** | | **Genotype - CpG** | | **CpG - Phenotype** | |
| --- | --- | --- | --- | --- | --- | --- | --- | --- | --- | --- | --- | --- | --- | --- |
| **Phenotype** | **SNP** | **Chr** | **Position (b37)** | **CpG** | **Position (b37)** | **CpG island info** | **Genomic Location** | **Gene** | **P-value** | **beta** | **P-value** | **beta** | **P-value** | **beta** |
| GSH | rs6750142 | 2 | 111923629 | cg07547695 | 111881075 | Shore | 5UTR | *BCL2L11* | 7.09E-04 | 42.21 | 1.24E-05 | -0.006 | 1.16E-08 | -2.0E-05 |
| TGSH | rs6750142 | 2 | 111923629 | cg07547695 | 111881075 | Shore | 5UTR | *BCL2L11* | 5.54E-04 | 44.75 | 1.24E-05 | -0.006 | 1.55E-08 | -1.9E-05 |
